# Improving Cancer Awareness and Knowledge in Johannesburg and iLembe Districts Through a Tailored Community-Based Educational Intervention: A Pilot Study

**DOI:** 10.3390/ijerph23070871

**Published:** 2026-07-03

**Authors:** Buhle Lubuzo, Usangiphile Buthelezi, Zamasomi Prudence Luvuno, Sithabisile Gugulethu Gigaba, Bridgette Goeieman, Wilbroda Hlolisile Chiya, Sibongile Ramotshela

**Affiliations:** 1 School of Nursing, University of KwaZulu-Natal, Durban 4041, South Africa; butheleziu@ukzn.ac.za (U.B.); luvunoz@ukzn.ac.za (Z.P.L.); gigabas@ukzn.ac.za (S.G.G.); chiyaw@ukzn.ac.za (W.H.C.); 2 Centre for Research in Health Systems, University of KwaZulu-Natal, Durban 4041, South Africa; 3 Genius Quality, Durban 4319, South Africa; 4 Afia Tai, Johannesburg 2197, South Africa; sisalehae47@gmail.com (B.G.); sramotshela@afiatai.org.za (S.R.)

**Keywords:** cancer awareness, community-based intervention, cancer education, South Africa, traditional health practitioners, c-CARE module, public health

## Abstract

**Highlights:**

**Public health relevance—How does this work relate to a public health issue?**

- Persistent inequities in cancer awareness, early detection, and access to screening and diagnostic services contribute to delayed diagnosis and preventable cancer mortality in low- and middle-income countries.
- This study addresses these inequities by assessing a culturally adapted, community-based cancer education intervention implemented among community-based cadres in underserved urban and rural South African settings.

**Public health significance—Why is this work of significance to public health?**

- The adapted c-CARE intervention was associated with significant improvements in awareness and knowledge across five cancers, with particularly large gains for multiple myeloma, a less recognised cancer associated with delayed diagnostic pathways.
- The findings show that culturally responsive cancer education can strengthen knowledge of cancer symptoms, risk factors, screening, diagnostic approaches, and supportive care among community-based actors positioned to influence health beliefs and help-seeking.

**Public health implications—What are the key implications or messages for practitioners, policy makers and/or researchers in public health?**

- Integrating structured cancer education into community health worker, faith-based, and traditional practitioner networks may strengthen community-level cancer literacy and support earlier recognition, referral, and engagement with health services.
- Future community-based cancer education programmes should combine factual knowledge with fear-reduction, myth correction, referral information, and stronger linkage to accessible screening, diagnostic, palliative, and spiritual care services.

**Abstract:**

Cancer remains a growing public health concern in South Africa, particularly in underserved communities where disparities in awareness and access to care contribute to delayed diagnosis. This study evaluated the impact of a culturally tailored educational intervention based on an adapted Cancer-Community Awareness Access Research and Education (c-CARE) module in Johannesburg and iLembe districts. A pilot study using a quasi-experimental pre–post design was conducted to assess changes in knowledge and attitudes among 210 traditional health practitioners, community health workers, and faith-based leaders. Structured surveys measured awareness of multiple myeloma, prostate, lung, breast, and cervical cancers. Data were captured in REDCap and analyzed using SPSS version 30. Significant improvements in knowledge were observed across all cancers. Awareness of lung cancer increased from 74.3% to 96.7%, multiple myeloma from 26.7% to 96.7%, prostate cancer from 52.5% to 98.3%, breast cancer from 93.4% to 98.7%, and cervical cancer from 84.8% to 96.0%. Participants demonstrated improved understanding of screening modalities and risk factors, including tobacco-related harms. Despite these gains, screening-related fears remained evident. These findings demonstrate that contextually adapted, community-based training can strengthen cancer literacy and support early detection strategies in underserved settings.

## 1. Introduction

Cancer represents a growing global public health challenge, with an estimated 19.3 million new cases and nearly 10 million deaths reported worldwide in 2020 [1]. In South Africa, as in other low- and middle-income countries (LMICs), the growing burden of cancer is shaped by demographic, structural, and health system factors, including population ageing, late diagnosis, limited access to quality healthcare, constrained diagnostic and treatment capacity, and shortages of specialised oncology personnel [2,3,4]. The global cancer observatory data indicate that breast, cervical, prostate, lung, and colorectal cancers account for a substantial proportion of the country’s cancer burden, with incidence patterns shaped by demographic transitions, improved detection, and persistent inequities in access to prevention, screening, diagnosis, and treatment services [5,6]. These constraints contribute to late-stage presentation and poorer survival outcomes, particularly in underserved and rural communities [7].

It is well established that improving cancer outcomes requires not only strengthening diagnostic and treatment capacity, but also improving public awareness, cancer literacy, early symptom recognition, and timely engagement with health services [8]. Limited public awareness and low cancer literacy can delay help-seeking, particularly when people are unable to recognise symptoms, understand risk factors, or navigate available services [9,10]. Evidence from LMIC settings suggests that improving community-level knowledge of cancer symptoms, risk factors, and available services can facilitate earlier recognition of symptoms, increase engagement with health services, and support timely diagnosis [11,12]. However, cancer education is unlikely to improve early detection if it is disconnected from the cultural, social, and health system realities of the populations it aims to serve. Educational interventions are therefore most likely to improve knowledge and promote early detection behaviours when they are contextually relevant, culturally responsive, and delivered through trusted community structures [11,13,14].

In South Africa, primary healthcare (PHC) remains central to cancer prevention, early detection, referral, and ongoing support, especially in rural and underserved communities [15]. However, PHC services often operate under resource constraints, while communities continue to experience unequal access to health education, screening, diagnostic services, and timely referral [16]. The psychosocial, palliative, and spiritual dimensions of cancer further complicate these challenges. A cancer diagnosis may be associated with fear, stigma, anxiety, depression, existential distress, and concerns about death, all of which can affect quality of life, treatment adherence, and engagement with care [17,18,19]. Yet these dimensions of care are often under-integrated into routine cancer services, particularly in resource-constrained settings.

Community-based educational initiatives offer a strategy for addressing gaps in cancer awareness and early detection in underserved settings. These approaches may be particularly effective when they engage trusted community actors who influence health beliefs, help-seeking behaviour, and treatment decisions [20,21]. In many African settings, health-seeking behaviour is shaped by a pluralistic healthcare landscape in which individuals engage with biomedical, traditional, religious, and community-based sources of support [22,23,24]. Traditional health practitioners (THPs), community health workers (CHWs), and faith-based leaders (FBLs) often serve as trusted sources of health information, guidance, emotional support, and referral. Rather than constituting barriers to biomedical care, these actors can be engaged as important partners in promoting cancer awareness, correcting misconceptions, supporting culturally appropriate communication, and strengthening linkages between communities and the formal health system [25,26].

The Cancer–Community Awareness Access Research and Education (c-CARE) programme, developed by the Georgia Cancer Center in the United States, provides one example of a community-based cancer education model. The programme has been used to improve cancer awareness among underserved populations by equipping community leaders and advocates with knowledge about cancer prevention, risk factors, early detection, screening, and treatment options [27,28,29]. By leveraging the trust and influence of community-based actors, c-CARE aims to improve cancer literacy, reduce stigma and misconceptions, and promote timely access to healthcare services [27,28,29]. However, transferring such interventions across contexts requires careful adaptation to ensure alignment with local epidemiological profiles, health system structures, referral pathways, service availability, language, cultural norms, and community beliefs.

Although implementation science and intervention adaptation frameworks provide guidance for the systematic adaptation of evidence-based interventions [30], there is limited empirical evidence on how cancer education programmes developed in high-income settings can be adapted and implemented in LMIC contexts, particularly in sub-Saharan Africa. There is also limited evidence on whether adapted cancer education interventions can improve awareness and knowledge among community-based cadres who operate across formal, traditional, and faith-based systems of care. This gap is important because these cadres are often well positioned to influence community understanding of cancer, support early recognition of symptoms, address misconceptions, and link individuals to formal health services.

Building on this model, the present study adapted the Georgia Cancer Center’s c-CARE module for implementation in two South African districts: Johannesburg in Gauteng Province and iLembe District in KwaZulu-Natal Province. These sites were purposively selected to reflect diverse socio-demographic and health system contexts, including urban and peri-urban populations in Johannesburg and more rural and resource-constrained communities in iLembe. The adaptation process involved contextualising the original c-CARE educational materials to align with the South African health system, local cancer epidemiology, available referral pathways, and the cultural and social contexts of the target communities. This included modifying cancer-specific content, incorporating locally relevant examples of risk factors and health services, and adding content on palliative care and spirituality in cancer care [31].

This study therefore assessed changes in cancer awareness and knowledge following participation in a culturally adapted c-CARE educational intervention among community-based cadres in Johannesburg and iLembe. The intervention focused on five cancers: breast, cervical, prostate, and lung cancers, selected due to their high burden in South Africa, and multiple myeloma, which, although less common, is frequently diagnosed at advanced stages due to non-specific symptoms and prolonged diagnostic pathways [32,33]. By targeting THPs, CHWs, and FBLs, this study sought to assess whether a contextually adapted educational intervention could improve cancer awareness and knowledge in underserved settings, while also generating insights into the implementation of culturally responsive cancer education in LMIC contexts.

## 2. Materials and Methods

### 2.1. Study Settings

The study was conducted in two regions of SA: Johannesburg, an urban centre in Gauteng, and iLembe District, a rural area in KwaZulu-Natal (KZN). These sites were purposively selected to capture variation in socio-demographic profiles, healthcare access, and service delivery contexts, including urban/peri-urban settings in Johannesburg and predominantly rural communities in iLembe.

### 2.2. Study Design

A community-based educational intervention was implemented in two districts, Johannesburg and iLembe, using a quasi-experimental design to evaluate the association of the adapted c-CARE module on cancer awareness and knowledge among non-healthcare professionals. This study utilized a pre-and post-intervention design to evaluate changes associated with a tailored community-based cancer awareness education program. The intervention involved training participants using an adapted version of the Georgia Cancer Center’s c-CARE module. Structured surveys were administered to assess changes in participants’ cancer-related knowledge, attitudes, and awareness before and after the training.

### 2.3. Study Population and Size

The study population consisted of THP, CHWs, Outreach Team leaders (OTLs—professional or enrolled nurses responsible for supervising CHWs), and Faith-Based Leaders (FBLs), who were identified as key community figures who are frequently consulted for health advice in their respective communities. Participants were recruited using a purposive sampling approach to ensure inclusion of key community-based stakeholders actively engaged in health and social support roles. CHWs were recruited through local health facilities, THPs through the Traditional Leadership Council, and FBLs through the South African Chaplain Association. Inclusion criteria included: (i) active involvement in community engagement or health-related activities; (ii) willingness to participate in the training and follow-up assessments; and (iii) availability during the entire training period. Exclusion criteria included individuals who were not actively engaged in community-based health or social support roles, those unable to provide informed consent, and participants who were unavailable for the full duration of the training. A formal sample size calculation was not conducted, as this study was designed as a pilot intervention. The sample size was therefore determined pragmatically based on feasibility, participant availability, and implementation capacity across the two study sites. The primary aim was to generate preliminary data to inform the design and sample size estimation of a future adequately powered study. A total of 210 participants were recruited for the training sessions. This included 151 females (71.9%) and 59 males (28.1%), with a median age of 48 years for females and 51 years for males.

### 2.4. Intervention

The intervention comprised a series of educational sessions based on the adapted c-CARE module, which was tailored to the local South African context. The adaptation process was informed by Card et al.’s seven-step framework for adapting evidence-based interventions [31,34]. This involved a structured, participatory process that included stakeholder consultation, systematic review of the original c-CARE programme materials, identification of contextual mismatches (including linguistic, cultural, and health system differences), and iterative modification of content and delivery approaches. The process incorporated input from community health workers, traditional health practitioners, faith-based leaders, and Department of Health representatives through a series of consultative meetings, and resulted in the integration of locally relevant epidemiological data, translation of materials into local languages, alignment with South African referral pathways, and inclusion of additional modules on palliative care and spirituality, while preserving the core components of the original intervention. Further details of the adaptation process are described elsewhere [31]. The training was designed to improve knowledge on cancer prevention, early detection, risk factors, and treatment options, with a focus on five types of cancer: cervical, lung, breast, prostate, and multiple myeloma. Additional content addressed palliative care, as well as linkage to local resources for cancer treatment and care. The content was delivered through interactive workshops utilising the adapted c-Care Manual and engaging in group activities. Training sessions were conducted in local languages, ensuring accessibility for participants with varying literacy levels. The training focused on empowering participants to disseminate cancer awareness information within their communities. Training was delivered over five consecutive days in each district. Each training session lasted approximately 6–8 h, resulting in a total training exposure of approximately 30–40 h per participant.

### 2.5. Data Collection

Data were collected using structured surveys administered before and after the training intervention to assess changes in participants’ knowledge and attitudes toward cancer. The survey instrument was adapted from previously published cancer awareness and education tools used in the c-CARE programme and related studies (Table S1). The instrument was contextually adapted for the South African setting, including modifications to terminology, examples, and content to ensure relevance and clarity. The instrument included domains on: (i) cancer awareness; (ii) knowledge of symptoms and risk factors; (iii) screening knowledge; and (iv) attitudes and perceptions toward cancer and screening. The primary outcome was the overall change in cancer awareness or knowledge pre- and post-training. The secondary outcomes were changes in symptom recognition, screening knowledge, and attitudes toward cancer and screening. Surveys were administered in paper format during the in-person training sessions, and the data were subsequently entered into REDCap, a secure web application for managing online surveys and databases. Mentoring sessions were conducted immediately following the intervention. Three months later, mentoring sessions and follow-up sessions were conducted to assess retention of cancer knowledge, ongoing awareness, and the extent to which participants had shared the information within their communities.

### 2.6. Data Analysis

Data were analysed using SPSS Version 30 (IBM Corp., Armonk, NY, USA) [35]. Categorical variables are presented as frequencies and percentages. Given the repeated measures design, in which the same participants (*n* = 210) completed identical survey instruments before and after the c-CARE educational intervention, paired categorical data were compared using McNemar’s test. This test is appropriate for assessing within-participant changes in binary outcomes across two time points [36]. Variables with more than two response categories, including multiple-choice knowledge items, were dichotomised into correct versus incorrect responses prior to inferential analysis. Incorrect responses included wrong responses and “don’t know” responses. For “select all that apply” items, each response option was treated as a separate binary variable, indicating whether the option was selected or not selected.

Socio-demographic characteristics are presented descriptively, stratified by sex, with no inferential testing performed on baseline characteristics. Cancer-specific awareness and knowledge outcomes, attitudes toward cancer and screening, and knowledge of palliative and spiritual care were analysed using McNemar’s test to evaluate changes from pre- to post-intervention. Statistical significance was set at *p* < 0.05 using two-tailed tests. Results are reported for the full study sample, except where specific items were only applicable to participants who had heard of a particular cancer, in which case the relevant denominator is reported in the corresponding table. No adjustment for multiple comparisons was applied; therefore, *p*-values are interpreted in the context of exploratory hypothesis testing.

### 2.7. Ethical Considerations

The study received ethical approval from the Biomedical Research Ethics Committee (BREC) of the University of KwaZulu-Natal (Approval No.: BREC/00004890/2022) and the Research Committee of the Johannesburg District (NHRD Reference No.: GP_202211_070). Additionally, permission was granted from the Province of KwaZulu-Natal Provincial Health Research and Ethics Committee, as well as support obtained from the iLembe Health District. Informed consent was obtained from all participants before their inclusion in the study. To ensure confidentiality, personal identifiers were not used in the computer-based survey or during workshops; instead, each participant was assigned a unique Participant Identification (PID) number. Participants were assured that their responses would remain confidential, and their participation was entirely voluntary, with the option to withdraw at any time.

## 3. Results

This study assessed pre- and post-intervention changes in cancer awareness, knowledge, attitudes, and perceptions among community-based cadres who participated in an adapted c-CARE educational intervention in Johannesburg and iLembe districts. The results are presented in line with the study objectives and survey domains. First, we describe the distribution of cadres trained across KwaZulu-Natal and Gauteng provinces, followed by the socio-demographic characteristics of participants. We then report changes in cancer awareness across the five cancer types included in the intervention: multiple myeloma, prostate, lung, breast, and cervical cancer. Thereafter, findings are presented on screening and diagnostic knowledge, knowledge of cancer risk factors, knowledge of cancer signs and symptoms, and attitudes and perceptions toward cancer and screening. Palliative care and spirituality are reported as secondary outcomes, since they were included as additional components of the adapted module.

### 3.1. Cadres Trained by Province

**Figure 1:**
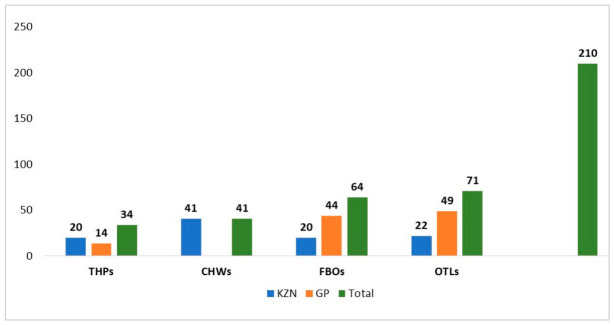
Cadres trained by province.

A total of 210 community-based cadres were trained across KwaZulu-Natal and Gauteng provinces. In KwaZulu-Natal, participants included 20 traditional health practitioners, 41 community health workers, 20 faith-based leaders, and 22 outreach team leaders. In Gauteng, the training included 14 traditional health practitioners, 44 faith-based leaders, and 49 outreach team leaders. Overall, the trained participants comprised 34 traditional health practitioners, 41 community health workers, 64 faith-based leaders, and 71 outreach team leaders. Figure 1 presents the distribution of trained cadres by province.

### 3.2. Participant Characteristics

The study included 210 participants, of whom 59 were male and 151 were female (Table 1). The median age was 51 years among men (range: 24–76 years) and 48 years among women (range: 23–73 years). Most participants were between 40 and 59 years of age, with the largest proportion of men in the 50–59-year age group and women in the 40–49-year age group. Christianity was the most commonly reported religion among both men and women, reported by 72.9% and 88.7% of participants, respectively. Most participants had attained at least secondary education. Secondary education was reported by 47.5% of men and 47.7% of women, while tertiary education was reported by 27.1% of men and 43.1% of women. Employment status differed by sex, with full-time employment reported more frequently among women (57.0%) than men (18.6%). Conversely, unemployment was reported by 28.8% of men compared with 14.6% of women. Public health facilities were the most commonly reported first point of contact when seeking care, reported by 83.1% of men and 72.2% of women. Other sources of care included private health facilities, traditional health practitioners, spiritual healers, and other providers (Table 1).

### 3.3. Cancer Awareness Before and After the Intervention

**Figure 2:**
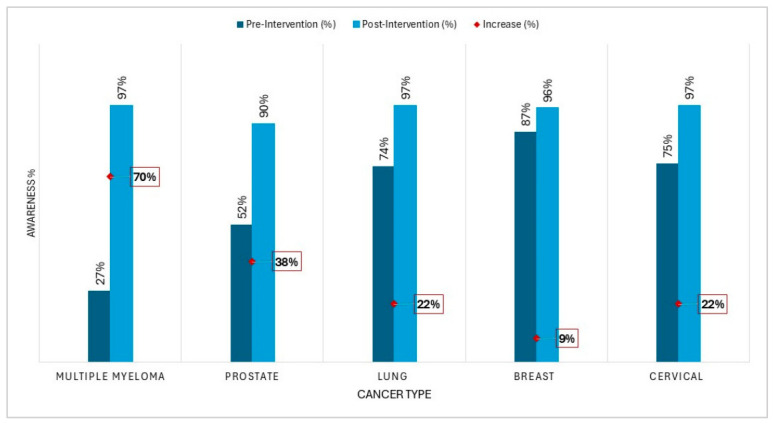
Cancer awareness before and after the c-CARE intervention.

Awareness of all five cancer types increased following the adapted c-CARE training (Figure 2). The greatest improvement was observed for multiple myeloma, where awareness increased from 26.7% before the intervention to 96.7% after the intervention (*p* < 0.001). Awareness of prostate cancer also increased substantially, from 52.5% pre-intervention to 98.3% post-intervention (*p* < 0.001). Similarly, awareness of lung cancer increased from 74.3% to 96.7% (*p* < 0.001). Although baseline awareness was already relatively high for breast and cervical cancers, awareness increased further from 87.1% to 95.7% and from 74.8% to 96.7%, respectively. Overall, the findings indicate that the adapted c-CARE training improved participants’ awareness of all five cancer types, with the largest gains observed for cancers that had lower levels of awareness at baseline.

### 3.4. Screening and Diagnostic Knowledge

**Figure 3:**
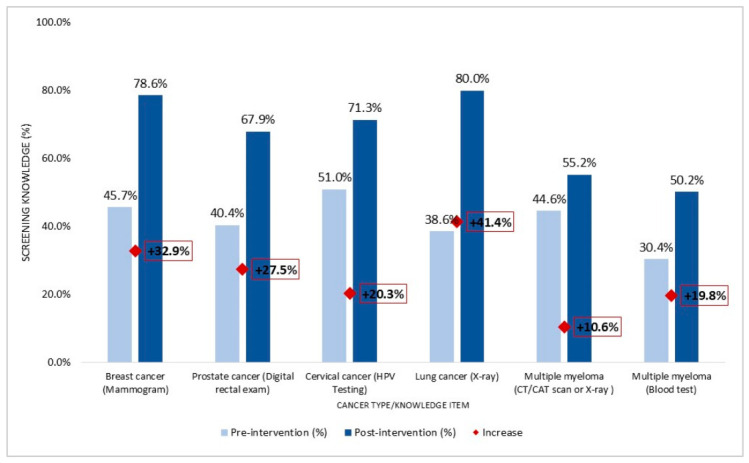
Screening knowledge before and after the intervention.

Screening and diagnostic knowledge improved across most cancer types following the adapted c-CARE training (Figure 3). For breast cancer, the proportion of participants who correctly identified mammography as the recommended screening test increased from 45.7% pre-intervention to 78.6% post-intervention (*p* < 0.001). Knowledge of a common prostate cancer screening method also improved, with correct identification of digital rectal examination increasing from 40.4% to 67.9% (*p* = 0.022).

For cervical cancer, knowledge of the appropriate HPV vaccination target group improved significantly, with correct identification of girls aged 10 years in Grade 5 increasing from 51.0% to 71.3% (*p* = 0.002). Lung cancer screening knowledge also improved, with identification of X-ray as a recommended screening test increasing from 38.6% to 80.0%, although the difference was not statistically significant (*p* = 0.062). For multiple myeloma, improvements were observed in diagnostic knowledge. Recognition of CT/CAT scans or X-rays as tests that can assist in diagnosis increased from 44.6% to 55.2% (*p* < 0.001), while recognition of blood tests increased from 30.4% to 50.2% (*p* < 0.001). Overall, the findings indicate improved screening and diagnostic knowledge following the intervention.

### 3.5. Knowledge of Risk Factors

Knowledge of cancer risk factors improved following the intervention, although gains varied across cancer types and specific knowledge items (Table 2). General understanding of cancer risk factors improved significantly, with the proportion of participants correctly identifying cancer risk factors as behavioural, environmental, genetic, and biological increasing from 14.8% pre-intervention to 45.7% post-intervention (*p* < 0.001). This suggests improved recognition that cancer risk is multifactorial rather than limited to a single cause such as genetics or family history.

For lung cancer, knowledge related to tobacco-related risk also improved. The proportion of participants who correctly identified nicotine as the cigarette ingredient that does not directly cause lung cancer increased from 6.2% to 43.3% (*p* = 0.021), while the proportion of participants who reported that they did not know declined from 44.8% to 9.5%. Participants’ understanding of the harms of electronic cigarettes also improved descriptively, with those identifying e-cigarettes as just as harmful as smoking cigarettes increasing from 35.7% to 77.6%; however, this change was not statistically significant (*p* = 0.171).

For cervical cancer, knowledge of several risk factors improved significantly. Recognition of HPV or HIV as risk factors increased from 56.1% to 84.1% (*p* < 0.001), while recognition of smoking or tobacco use increased from 42.0% to 54.1% (*p* < 0.001). The proportion of participants identifying having many different sexual partners as a risk factor also increased from 65.0% to 78.3% (*p* = 0.001). However, some responses also suggest persistent uncertainty or possible confusion, including increased selection of family history and oral contraception as risk factors. For prostate cancer, correct identification of frequent or excessive sweating in the groin area as not being a prostate cancer symptom increased from 19.3% to 38.5%, although this change was not statistically significant (*p* = 0.414). Among participants who had heard of multiple myeloma, correct recognition of the C.R.A.B. acronym increased from 25.0% to 43.3% (*p* = 0.013), indicating improved knowledge of key clinical features associated with multiple myeloma. No dedicated breast cancer risk-factor item was available in the dataset. The overall findings indicate improved risk-factor knowledge after the intervention, but also highlight areas where misconceptions and incomplete understanding remained

### 3.6. Knowledge of Cancer Signs and Symptoms

Knowledge of cancer signs and symptoms generally improved following the intervention, although the level of improvement varied across cancer types (Table 3). For lung cancer, the proportion of participants who correctly identified “trouble breathing, weight gain, cough and voice changes” as the statement that was not correct increased from 31.4% pre-intervention to 59.6% post-intervention (*p* = 0.020), indicating improved recognition of lung cancer symptom patterns. Missing responses also declined from 31.4% to 5.7%, suggesting greater confidence in responding to this item after the training.

For cervical cancer, knowledge of symptoms improved across all listed items. The proportion of participants identifying abnormal or smelly vaginal discharge increased from 67.5% to 86.0% (*p* < 0.001), while recognition of pain during sex increased from 63.1% to 86.6% (*p* < 0.001). Awareness of heavier or longer menstrual periods increased from 61.8% to 78.3% (*p* = 0.005), and recognition of vaginal bleeding after menopause increased from 58.6% to 79.0% (*p* < 0.001). Improvements were also observed for ongoing lower back pain, unexplained weight loss, and irregular vaginal bleeding. However, the increase in the selection of bloody stools may suggest some residual confusion about symptoms that are less specific to cervical cancer.

For breast cancer, correct identification of tingling or itching in an area of the breast as the statement that was not correct increased from 42.4% to 57.1%, although this change did not reach statistical significance (*p* = 0.070). For prostate cancer, correct identification of frequent or excessive sweating in the groin area as the statement that was not correct increased from 19.3% to 38.5%, but this change was also not statistically significant (*p* = 0.414). Among participants who had heard of multiple myeloma, correct recognition of the C.R.A.B. acronym as calcium, renal failure, anaemia, and bone lesions increased from 25.0% to 43.3% (*p* = 0.013). These findings suggest improved symptom knowledge after the intervention, particularly for cervical cancer, lung cancer, and multiple myeloma, while knowledge gains for breast and prostate cancer symptom items were more modest.

### 3.7. Attitudes and Perceptions Toward Cancer and Screening

The adapted c-CARE training produced statistically significant shifts across several cancer-related attitude domains, reflecting meaningful reductions in fatalism, misconceptions, and avoidance, as well as reinforcement of positive health-seeking attitudes.

#### 3.7.1. Fear and Avoidance of Cancer Screening

At baseline, a large majority of participants (77.1%) agreed that most people avoid cancer screening out of fear of receiving a positive diagnosis. Following the intervention, this proportion increased significantly to 92.4% (*p* = 0.005), indicating that participants had a heightened awareness of screening avoidance as a widespread psychological barrier. This finding suggests that the training was effective not only in conveying factual content but in sensitising participants to the emotional and psychosocial obstacles that deter communities from engaging with cancer screening services. Similarly, the proportion who agreed that most people prefer not to know if they have cancer increased from 50.5% pre-intervention to 71.0% post-intervention (*p* = 0.005), further underscoring the improved recognition of avoidance behaviour as a significant community-level challenge.

#### 3.7.2. Fatalism and Perceived Agency

Two attitude items probed the degree to which participants held fatalistic beliefs about cancer. Prior to the intervention, 21.0% of participants agreed with the statement “If I get cancer, it was meant to be,” reflecting a fatalistic orientation that may undermine engagement with prevention and early detection. Post-intervention, this proportion decreased modestly to 18.6%, with a significant shift in the distribution of responses overall (*p* < 0.001), driven primarily by an increase in disagreement from 58.1% to 71.4%. Similarly, agreement with the statement “There is not much that a person can do to lower their chances of getting cancer” decreased marginally from 19.5% to 20.5%, but again the overall response distribution shifted significantly (*p* < 0.001), with disagreement increasing from 54.3% to 71.9% and uncertainty declining substantially from 12.9% to 3.3%. Together, these findings suggest the intervention was effective in challenging fatalistic beliefs and strengthening a sense of personal agency in cancer prevention, which is critical for motivating health-seeking behaviour in community settings.

#### 3.7.3. Value of Regular Cancer Screening

The vast majority of participants recognised at baseline that regular cancer checks help detect cancer when it is easier to treat (89.5%), and this proportion increased further to 96.2% following the intervention (*p* = 0.012). The high endorsement of this statement post-intervention, combined with the significant reduction in uncertainty and non-response, indicates that the training successfully consolidated positive attitudes towards the value of early detection across all participants.

#### 3.7.4. Cancer and Mortality Associations

At baseline, nearly three-quarters of participants (72.4%) agreed that when people think of cancer, they automatically associate it with death, and this proportion increased to 84.3% post-intervention, though this change did not reach statistical significance (*p* = 0.479). While this item did not change significantly, the persistently high agreement across both time points highlights the enduring conflation of cancer with mortality in community consciousness, which is a deeply entrenched perception that single-session educational interventions may be insufficient to alter, and which warrants ongoing attention in community health communication efforts.

#### 3.7.5. Misconceptions About Cancer Causation and Spread

Two items assessed specific misconceptions. Disagreement with the statement “Exposing cancer to air during surgery causes it to spread faster”. The correct response increased significantly from 45.7% to 63.8% (*p* < 0.001), while uncertainty and non-response declined markedly (from 41.0% combined to 17.6%). This indicates meaningful correction of a common and potentially harmful surgical misconception following the intervention. Regarding the statement “It seems like everything causes cancer,” disagreement increased from 44.8% to 61.9% (*p* = 0.004), accompanied by a substantial reduction in “don’t know” responses from 32.4% to 12.9%. This shift suggests that participants developed a more discerning understanding of cancer aetiology, moving away from a generalised sense of helplessness or confusion about causation toward a more structured view of identifiable risk factors.

#### 3.7.6. Comfort Discussing Medical History

Prior to the intervention, only 15.2% of participants agreed that most people are comfortable discussing their medical history, with 43.8% disagreeing and a notable proportion uncertain (17.6%) or unable to respond (23.3%). Post-intervention, the distribution shifted substantially (*p* = 0.089), with disagreement increasing to 67.1% and uncertainty halving to 5.2%, although the change did not reach conventional statistical significance. The overall pattern nonetheless suggests a sharpening of awareness that medical history disclosure remains a barrier in community contexts, which is an attitude that is relevant to the uptake of cancer risk assessment and screening.

Overall, the attitude findings suggest that the intervention did not produce a simple shift from negative to positive perceptions, but rather revealed a more complex pattern. Participants showed stronger recognition of prevention, early detection, and the possibility of reducing cancer risk, as reflected in increased disagreement with fatalistic statements and greater support for regular cancer checks. At the same time, fear of screening and preference not to know one’s cancer status increased, suggesting that improved awareness may also have made the seriousness of cancer more visible. This apparent contradiction is important in real-world community settings, where increased knowledge may not immediately translate into reduced fear or greater readiness to screen. Instead, it may first heighten awareness of the emotional, social, and psychological barriers that shape cancer-related decision-making.

### 3.8. Palliative Care and Spirituality

#### 3.8.1. Palliative Care and Spiritual Care (Secondary Outcomes)

Palliative care and spirituality were included as additional components of the adapted c-CARE module, and changes in these domains are reported as secondary outcomes.

#### 3.8.2. Palliative Care Knowledge

Prior to the intervention, foundational knowledge of palliative care was variable across the sample. Misconceptions were common: approximately one quarter of participants (24.8%) incorrectly characterised palliative care as care for people who are dying only, and 7.1% described it as care for children only. Following the intervention, both misconceptions were significantly reduced, to 15.7% (*p* = 0.013) and 2.4% (*p* = 0.031) respectively. Conversely, recognition of accurate descriptions of palliative care improved markedly. Identification of palliative care as care for patients and their families increased from 48.6% to 68.6% (*p* < 0.001), and recognition of palliative care as encompassing the physical, social, emotional, and spiritual needs of patients increased from 66.2% to 84.3% (*p* < 0.001).

Knowledge of the multidisciplinary composition of the palliative care team also improved following the intervention. Recognition of social workers and social auxiliary workers as team members increased from 73.3% to 84.3% (*p* = 0.002), and recognition of chaplains and spiritual counsellors increased from 65.2% to 86.7% (*p* = 0.002). Notably, recognition of community health care workers as members of the palliative care team improved significantly, from 69.1% to 84.8% (*p* < 0.001), which is particularly relevant in the South African community health care context. Recognition of doctors and nurses as team members was already high at baseline (72.9% and 73.3% respectively) and showed no statistically significant change post-intervention (*p* = 0.341 and *p* = 0.322 respectively).

Regarding the role of community health care workers in palliative care, significant improvements were observed in two domains. Recognition that community health care workers can treat pain and other distressing symptoms increased from 42.4% to 54.8% (*p* = 0.003), and recognition of their role in identifying patients who need palliative care increased from 65.7% to 79.5% (*p* < 0.001). Knowledge of their role in referring patients to a healthcare worker showed a trend toward improvement (71.9% to 79.5%), though this did not reach statistical significance (*p* = 0.064). Knowledge of their role in monitoring medication adherence was already relatively high at baseline (79.5%) and did not change significantly post-intervention (*p* = 0.174).

#### 3.8.3. Spiritual Care Knowledge and Attitudes

Significant improvements were observed across multiple dimensions of spiritual care knowledge following the intervention. Among statements about the nature of spirituality, the proportion of participants who agreed that spirituality is important to cancer patients increased from 44.3% to 55.2% (*p* = 0.014), and agreement that spirituality improves the quality of life of cancer patients increased from 58.1% to 72.4% (*p* < 0.001). Recognition that unaddressed spiritual needs can result in suffering increased substantially, from 34.3% to 57.6% (*p* < 0.001), and agreement that the root cause of many difficulties people face can be spiritual increased from 55.7% to 72.4% (*p* < 0.001). Recognition that cancer patients have spiritual needs also improved, from 59.5% to 68.6% (*p* = 0.037).

Knowledge of spiritual care practices was similarly enhanced. Recognition of listening as a spiritual care practice increased from 69.1% to 84.8% (*p* < 0.001), and recognition of screening for spiritual needs increased from 60.0% to 71.0% (*p* = 0.009). Correct identification of referring patients to a spiritual counsellor or chaplain as a spiritual care practice increased from 70.0% to 81.9% (*p* = 0.001), and recognition of providing space for meditation and prayer improved from 68.6% to 78.1% (*p* = 0.013). Identifying patients’ resources for hope as a spiritual care practice also improved significantly, from 61.9% to 72.9% (*p* = 0.008).

With respect to the rights and scope of spiritual care, the proportion of participants who agreed that every patient has a right to receive spiritual care increased from 84.8% to 92.9% (*p* = 0.012), and recognition that spiritual care helps cancer patients who are in pain increased from 43.8% to 63.3% (*p* < 0.001). Agreement that community health care workers can pray with any patient when asked increased from 57.1% to 69.1% (*p* = 0.003), and recognition that spiritual care is part of holistic, patient-centred care increased from 54.3% to 68.1% (*p* = 0.001). The proportion who agreed that spiritual care may help patients find meaning in life showed a positive trend (71.4% to 78.6%), though this did not reach statistical significance (*p* = 0.058).

Taken together, the secondary outcome data suggest that the palliative and spiritual care components of the adapted c-CARE module were effective in addressing gaps in knowledge and correcting misconceptions within this community health care worker cohort. These findings underscore the value of integrating palliative and spiritual care content into community-based cancer education programmes, particularly in contexts where community health care workers serve as a critical interface between patients and the formal health system.

## 4. Discussion

This pilot study found that participation in the adapted c-CARE educational intervention was associated with substantial improvements in cancer awareness, symptom and risk-factor knowledge, screening-related knowledge, and understanding of palliative and spiritual care among community-based cadres in Johannesburg and iLembe. Improvements were observed across all five cancers included in the intervention, with the largest gains seen for multiple myeloma and prostate cancer, where baseline awareness was comparatively low. These findings suggest that a culturally adapted, community-based cancer education programme can improve cancer literacy among non-specialist actors who are already embedded in communities. However, the findings also show that improved knowledge did not translate into uniformly positive attitudes. Screening-related fear and preference not to know one’s cancer status persisted or increased after training, highlighting that cancer education may improve awareness while also making the emotional and social consequences of diagnosis more visible.

### 4.1. Cancer Awareness and Community-Based Cancer Literacy

The large improvements in awareness across all cancer types are consistent with evidence that structured, community-based education can strengthen cancer literacy, particularly where interventions are tailored to the language, social realities, and health system context of participants [11,14,26]. The substantial improvement in multiple myeloma awareness is particularly important because this cancer is often less visible in public health education than breast, cervical, lung, and prostate cancers [32,33]. In this study, the low baseline awareness of multiple myeloma created greater room for change, while breast and cervical cancer showed smaller increases because awareness was already relatively higher before the intervention. This pattern suggests that community-based education may be especially valuable for cancers that are under-recognised, have non-specific symptoms, or are less commonly included in public health messaging. It also reinforces the importance of broadening cancer education beyond the cancers that already receive greater public attention.

### 4.2. Screening, Symptoms, and Risk-Factor Knowledge

The intervention improved participants’ knowledge of screening and diagnostic approaches, including mammography for breast cancer, digital rectal examination for prostate cancer, HPV vaccination for cervical cancer, lung cancer screening knowledge, and diagnostic tests relevant to multiple myeloma. These findings are important because cancer awareness alone is unlikely to improve early detection unless people also understand when, where, and how to seek screening or diagnostic assessment. Evidence from LMIC settings shows that CHWs and other community-based actors often support cancer control through education, outreach, navigation, and referral, but their effectiveness depends on whether information is practical and linked to available services [26,37]. The improvement in risk-factor knowledge also suggests that the intervention helped participants move beyond narrow explanations of cancer causation toward a more multifactorial understanding involving behavioural, environmental, biological, and genetic risks. However, some areas of uncertainty remained, indicating that risk communication requires repeated reinforcement, particularly where biomedical concepts may compete with existing cultural explanations of illness.

### 4.3. Attitudes, Fear, and the Limits of Knowledge-Based Interventions

One of the most important findings was that improvements in knowledge were accompanied by persistent or increased fear of cancer screening and a preference not to know one’s cancer status. This should not be interpreted as intervention failure. Rather, it suggests that increased awareness may heighten recognition of cancer seriousness, uncertainty about treatment pathways, and fear of diagnosis. This is consistent with literature showing that cancer-related stigma, fear, fatalism, and concerns about disclosure can shape help-seeking even when awareness improves [38,39,40]. In community settings, knowledge is therefore necessary but insufficient. Educational interventions need to address emotional readiness, trust in referral pathways, perceived affordability, confidentiality, and confidence that screening will lead to timely and respectful care. This is especially relevant in underserved settings where people may associate diagnosis with suffering, delayed treatment, financial burden, or death. Future iterations of the c-CARE intervention should therefore include more explicit content on reducing fear, normalising screening, explaining what happens after referral, and supporting participants to communicate hope without minimising the seriousness of cancer.

### 4.4. Palliative Care, Spirituality, and Culturally Responsive Cancer Education

The improvements in palliative care and spiritual care knowledge are an important secondary finding. Participants showed a better understanding that palliative care is not limited to people who are dying, but includes support for patients and families and addresses physical, social, emotional, and spiritual needs. This aligns with global definitions of palliative care as holistic care that improves quality of life for patients and families facing life-threatening illness [41]. In the South African context, where faith-based leaders, traditional health practitioners, and CHWs often provide emotional, spiritual, and practical support, integrating palliative and spiritual care into cancer education is particularly relevant [42,43,44]. These findings suggest that the adapted c-CARE module did more than improve disease-specific knowledge; it also strengthened understanding of supportive care across the cancer pathway. This is important because community-based cadres may be well positioned to reduce misconceptions, provide psychosocial support, encourage early help-seeking, and link families to appropriate services.

### 4.5. Strengths

A key strength of this study is that it evaluated a culturally adapted intervention implemented among community-based cadres who are likely to influence health beliefs, help-seeking, and referral decisions in real-world settings. The inclusion of CHWs, outreach team leaders, traditional health practitioners, and faith-based leaders reflects the pluralistic nature of health-seeking in South Africa and recognises that cancer education does not only occur within formal facilities. The intervention was also delivered in local languages and adapted to local epidemiological, cultural, and health system realities, which likely improved relevance and acceptability. In addition, the pre–post design allowed the study to measure within-participant changes in knowledge and attitudes after the intervention. The use of McNemar tests was appropriate for paired categorical data and strengthened interpretation for pre- and post the intervention. Finally, the inclusion of palliative care and spirituality broadened the intervention beyond early detection alone and reflected a more holistic understanding of cancer care.

### 4.6. Limitations

This study should be interpreted in light of several limitations. First, as a pilot study, it did not include a control group, meaning that observed changes cannot be attributed to the intervention with the same certainty as in a controlled trial. Second, the sample was purposively selected and included community-based cadres from Johannesburg and iLembe; therefore, findings may not be generalisable to all districts or community groups in South Africa. Third, outcomes were measured immediately before and after training, which provides evidence of short-term knowledge change but not long-term retention, community dissemination, screening uptake, or diagnostic outcomes. Fourth, several variables were dichotomised into correct and incorrect responses, which supported statistical testing but may have reduced the nuance of participants’ knowledge and beliefs. Another limitation relates to the measurement tool used in this study. Although the survey instrument was adapted from previously published cancer awareness and education tools and modified to improve local terminology, language accessibility, and contextual relevance, the adapted version was not formally validated as a new instrument in this study population prior to implementation. As a result, its psychometric properties, including reliability and construct validity, were not established. This may have influenced how participants understood and responded to some items, particularly those involving multi-option knowledge questions and attitude statements. Therefore, the findings should be interpreted with caution, especially in relation to the magnitude of change observed across some knowledge and attitude domains. Finally, because no adjustment was made for multiple comparisons, *p*-values should be interpreted cautiously and in line with the exploratory nature of the study.

### 4.7. Implementation Implications

The findings have important implications for cancer control in underserved settings. They suggest that culturally adapted cancer education can strengthen cancer literacy among trusted community actors who are already positioned to support awareness, early recognition, and referral. However, the persistence of fear shows that future programmes should not focus only on information transfer. They should include counselling-oriented communication, myth correction, role-play on screening conversations, clear referral information, and stronger linkage between community education and accessible screening services. From an implementation perspective, c-CARE could be integrated into CHW training, outreach team supervision, faith-based health promotion, and partnerships with traditional health practitioners. However, its effectiveness will depend on whether education is connected to functioning screening and referral pathways. Raising awareness without strengthening service access may increase concern without improving early detection.

## 5. Conclusions

This study demonstrates that an adapted c-CARE educational intervention can improve cancer awareness and knowledge among community-based cadres in both urban and rural South African settings. The strongest gains were observed in cancers with lower baseline awareness, particularly multiple myeloma, while improvements in screening, risk-factor, palliative care, and spiritual care knowledge suggest broader benefits for community-based cancer literacy. However, the persistence of and increase in screening-related fear show that knowledge improvement alone is not enough. Cancer education interventions must also address emotional, cultural, and health system barriers that shape whether people act on knowledge. Future research should assess longer-term knowledge retention, community dissemination, screening uptake, referral completion, and the feasibility of integrating c-CARE into routine primary health care and community health systems.

## Figures and Tables

**Table 1:** Socio-demographic characteristics of participants.

	Male (*n* = 59)		Female (*n* = 151)	
**Age-Group**	**Number**	**Percent**	**Number**	**Percent**
20–29	2	3.4	5	3.3
30–39	10	17.0	39	25.8
40–49	16	27.1	49	32.5
50–59	17	28.8	38	25.2
60–69	8	13.6	17	11.3
70+	6	10.2	3	2.0
**Median (Range)**	**51 (24–76)**		**48 (23–73)**	
**Religion**				
Christian	43	72.9	134	88.7
Hindu	1	1.7	-	-
Muslim	7	11.9	13	8.6
African Traditional Religion	4	6.8	4	2.7
Other	4	6.8	-	-
**Education**				
Illiterate	3	5.1	3	2.0
Primary	12	20.3	11	7.3
Secondary	28	47.5	72	47.7
Tertiary	16	27.1	65	43.1
**Employment**				
Student (full or part-time)	3	5.1	2	1.3
Self-employed	15	25.4	19	12.6
Work Full-Time (36 h or more/week)	11	18.6	86	57.0
Work Part-Time (less than 36 h/week)	3	5.1	8	5.3
Retired and not employed.	3	5.1	10	6.6
Unemployed	17	28.8	22	14.6
Grant	7	11.9	4	2.7
**First point of contact when sick**				
Public health facility	49	83.1	109	72.2
Private health facility	5	8.5	23	15.2
Traditional Health Practitioner	4	6.8	8	5.3
Spiritual healer	-	-	6	4.0
Other: specify	1	1.7	1	0.7
Unknown	-	-	4	2.7

**Table 2:** Knowledge of cancer risk factors before and after the intervention.

Variable	Pre-Intervention Frequency	Pre-Intervention %	Post-Intervention Frequency	Post-Intervention %	*p*-Value
**General Cancer Risk Factors (*n* = 210)**					
***Which of the following best describes cancer risk factors?***					
**Genetic and family history only**	89	42.4	74	35.2	<0.001
**Behavioural, environmental,** **genetic and biological (correct)**	31	14.8	96	45.7	
**Biological, age, sex and race only**	2	1	3	1.4	
**Behavioural, smoking and** **occupation only**	77	36.7	36	17.1	
**Missing**	11	5.2	1	0.5	
**Total**	210	100	210	100	
**Lung Cancer (*n* = 210)**					
***Which one of the following cigarette ingredients does NOT cause Lung Cancer?***					
**Carbon monoxide**	24	11.4	38	18.1	
**Tar**	11	5.2	20	9.5	
**Nicotine (correct)**	13	6.2	91	43.3	0.021
**Methane**	9	4.3	27	12.9	
**Don’t know**	94	44.8	20	9.5	
**Missing**	59	28.1	14	6.7	
**Total**	210	100	210	100	
***Compared to smoking cigarettes, electronic cigarettes/e-cigs are:***					
**Much less harmful**	14	6.7	23	11	
**Less harmful**	10	4.8	7	3.3	
**Just as harmful (correct)**	75	35.7	163	77.6	0.171
**Not harmful at all**	2	1	4	1.9	
**Don’t know**	52	24.8	4	1.9	
**Missing**	57	27.1	9	4.3	
**Total**	210	100	210	100	
**Cervical Cancer (among participants who answered “Yes”, ** ***n*** ** = 157)**					
***Which of the following are risk factors (causes) of Cervical Cancer? (Select all that apply)***					
**Family members with cervical cancer—Yes**	75	47.8	84	53.5	<0.001
**Having HPV or HIV—Yes**	88	56.1	132	84.1	<0.001
**Smoking/tobacco use—Yes**	66	42	85	54.1	<0.001
**Use of oral contraception—Yes**	21	13.4	58	36.9	0.115
**Having many different sexual partners—Yes**	102	65	123	78.3	0.001
**Giving birth to many babies—Yes**	53	33.8	41	26.1	<0.001
*n* *	157		157		
**Prostate Cancer (among participants who answered “Yes”, *n* = 109)**					
***Choose the statement about the signs/symptoms of Prostate Cancer that is NOT correct.***					
**Difficult or painful urination or blood in the urine**	19	17.4	10	9.2	
**Slow or weak urinary stream/need to urinate more at night**	10	9.2	6	5.5	
**Frequent or excessive sweating in the groin area (correct—NOT)**	21	19.3	42	38.5	0.414
**Bone pain in hips, back or chest**	55	50.5	48	44	
**Missing**	4	3.7	3	2.8	
**Total**	109	100	109	100	
**Multiple Myeloma (subset among those who answered “Yes”: pre *n* = 56, post *n* = 203)**					
***The term C.R.A.B. stands for what, as it relates to Multiple Myeloma symptoms/risk indicators***					
**Cholesterol, Redness, Abscess, Bone Pain**	15	26.8	32	15.8	
**Cold, Redness, Abnormal Platelets, Bruising**	7	12.5	7	3.5	
**Calcium, Redness, Anemia, Bone Fracture**	10	17.9	70	34.5	
**Calcium, Renal Failure, Anemia, Bone Lesions (correct)**	14	25	88	43.3	0.013
**Missing**	10	17.9	6	3	
**Total (subset)**	56	100	203	100	

Note: *p*-values derived from McNemar test (paired pre- vs. post-intervention comparisons). No dedicated risk factor question was available for Breast Cancer in the dataset. * Multiple responses allowed; frequencies do not sum to *n*. Percentages are calculated using *n* as the denominator.

**Table 3:** Knowledge of cancer signs and symptoms before and after the intervention.

Variable	Pre-Intervention Frequency	Pre-Intervention %	Post-Intervention Frequency	Post-Intervention %	*p*-Value
**Lung Cancer (*n* = 210)**					
***Choose the statement about the signs of Lung Cancer that is NOT correct.***					
**Cough, bloody phlegm, chest pain** **and voice changes**	21	10	13	6.2	
**Bloody phlegm, weight loss, fatigue and voice changes**	20	9.5	24	11.4	
**Trouble breathing, weight gain, cough and voice changes (correct—NOT)**	66	31.4	118	56.2	0.020
**Trouble swallowing, frequent pneumonia, cough and fatigue**	37	17.6	43	20.5	
**Missing**	66	31.4	12	5.7	
**Total**	210	100	210	100	
**Cervical Cancer (among participants who answered “Yes”, *n* = 157)**					
***Which of the following are signs/symptoms of Cervical Cancer? (Select all that apply)***					
**Irregular vaginal bleeding—Yes**	129	82.2	132	84.1	0.002
**Ongoing lower back pain—Yes**	65	41.4	101	64.3	0.002
**Abnormal or smelly vaginal discharge—Yes**	106	67.5	135	86	<0.001
**Pain during sex—Yes**	99	63.1	136	86.6	<0.001
**Heavier or longer menstrual periods than normal—Yes**	97	61.8	123	78.3	0.005
**Vaginal bleeding after menopause—Yes**	92	58.6	124	79	<0.001
**Bloody stools—Yes**	33	21	66	42	<0.001
**Unexplained weight loss—Yes**	60	38.2	96	61.1	0.002
*n* *	**157**		**157**		
**Breast Cancer (** ***n*** ** = ** **210** **)**					
***Choose the statement about the signs of Breast Cancer that is NOT correct.***					
**New lump in breast or underarm; any change in size or shape of breast**	45	21.4	32	15.2	
**Thickening or swelling of breast; nipple discharge other than breast milk**	26	12.4	29	13.8	
**Tingling and/or itching in an area of the breast (correct—NOT)**	89	42.4	120	57.1	0.070
**Pain, redness or flaky skin in the nipple area or any area of the breast**	15	7.1	14	6.7	
**Missing**	35	16.7	15	7.1	
**Total**	210	100	210	100	
**Prostate Cancer (among participants who answered “Yes”, *n* = 109)**					
***Choose the statement about the signs/symptoms of Prostate Cancer that is NOT correct.***					
**Difficult or painful urination or blood in the urine**	19	17.4	10	9.2	
**Slow or weak urinary stream/need to urinate more often at night**	10	9.2	6	5.5	
**Frequent or excessive sweating in the groin area (correct—NOT)**	21	19.3	42	38.5	0.414
**Bone pain in hips, back or chest**	55	50.5	48	44	
**Missing**	4	3.7	3	2.8	
**Total**	109	100	109	100	
**Multiple Myeloma (subset among those who answered “Yes”: pre *n* = 56, post *n* = 203)**					
***The term C.R.A.B. stands for what, as it relates to Multiple Myeloma symptoms***					
**Cholesterol, Redness, Abscess, Bone Pain**	15	26.8	32	15.8	
**Cold, Redness, Abnormal Platelets, Bruising**	7	12.5	7	3.5	
**Calcium, Redness, Anemia, Bone Fracture**	10	17.9	70	34.5	
**Calcium, Renal Failure, Anemia, Bone Lesions (correct)**	14	25	88	43.3	0.013
**Missing**	10	17.9	6	3	
**Total (subset)**	56	100	203	100	

Note: *p*-values derived from the McNemar test (paired pre- vs. post-intervention comparisons). * Multiple responses allowed; frequencies do not sum to *n*. Percentages are calculated using *n* as the denominator.

## Data Availability

The original contributions presented in this study are included in the article and Supplementary Material. Further inquiries can be made to Admin@afiatai.org.za.

## Supplementary Materials

The following supporting information can be downloaded at: https://www.mdpi.com/article/10.3390/ijerph23070871/s1; Table S1: Baseline Survey (Pre- and Post) Questionnaire.

## Abbreviations

The following abbreviations are used in this manuscript:

SA	South Africa
KZN	KwaZulu-Natal
GP	Gauteng Province
LMICs	Low- and Middle-Income Countries
PHC	Primary Health Care
THP	Traditional Health Practitioner
CHW	Community Health Worker
OTL	Outreach Team Leader
FBL	Faith-Based Leader
c-CARE	Cancer-Community Awareness Access Research and Education
HPV	Human Papillomavirus
HIV	Human Immunodeficiency Virus
CRAB	Calcium, Renal failure, Anaemia, Bone lesions (multiple myeloma features)
REDCap	Research Electronic Data Capture
BREC	Biomedical Research Ethics Committee
BMSF	Bristol–Myers Squibb Foundation

## References

[1] Cao W., Chen H.D., Yu Y.W., Li N., Chen W.-Q. (2021). Changing profiles of cancer burden worldwide and in China: A secondary analysis of the global cancer statistics 2020. Chin. Med. J..

[2] Omotoso O., Teibo J.O., Atiba F.A., Oladimeji T., Paimo O.K., Ataya F.S., Batiha G.E.-S., Alexiou A. (2023). Addressing cancer care inequities in sub-Saharan Africa: Current challenges and proposed solutions. Int. J. Equity Health.

[3] Bamodu O.A., Chung C.C. (2024). Cancer Care Disparities: Overcoming Barriers to Cancer Control in Low- and Middle-Income Countries. JCO Glob. Oncol..

[4] Mwamba M., Lombe D.C., Msadabwe S., Bond V., Simwinga M., Ssemata A.S., Muhumuza R., Seeley J., Mwaka A., Aggarwal A. (2023). A Narrative Synthesis of Literature on the Barriers to Timely Diagnosis and Treatment of Cancer in Sub-Saharan Africa. Clin. Oncol..

[5] Finestone E., Wishnia J. (2022). Estimating the burden of cancer in South Africa. S. Afr. J. Oncol..

[6] Global Cancer Observatory (2022). Statistic at a Glance.

[7] Musekiwa A., Moyo M., Mohammed M., Matsena-Zingoni Z., Twabi H.S., Batidzirai J.M., Singini G.C., Kgarosi K., Mchunu N., Nevhungoni P. (2022). Mapping Evidence on the Burden of Breast, Cervical, and Prostate Cancers in Sub-Saharan Africa: A Scoping Review. Front. Public Health.

[8] Christiansen K., Buswell L., Fadelu T. (2023). A Systematic Review of Patient Education Strategies for Oncology Patients in Low- and Middle-Income Countries. Oncologist.

[9] Humphrys E., Burt J., Rubin G., Emery J.D., Walter F.M. (2019). The influence of health literacy on the timely diagnosis of symptomatic cancer: A systematic review. Eur. J. Cancer Care.

[10] Moodley J., Constant D., Mwaka A.D., Scott S.E., Walter F.M. (2020). Mapping awareness of breast and cervical cancer risk factors, symptoms and lay beliefs in Uganda and South Africa. PLoS ONE.

[11] Nnaji C.A., Kuodi P., Walter F.M., Moodley J. (2022). Effectiveness of interventions for improving timely diagnosis of breast and cervical cancers in low-income and middle-income countries: A systematic review. BMJ Open.

[12] Moodley J., Day S., Ras T., Ataguba J.E., Harries J., Jacobs R., Chirenje Z.M., Ghuza B., Payne A., Githaiga J.N. (2024). Mapping local evidence on early recognition and management of people with potential cancer symptoms: A narrative review. S. Afr. Health Rev..

[13] Zhang M., Sit J.W.H., Chan D.N.S., Akingbade O., Chan C.W.H. (2022). Educational Interventions to Promote Cervical Cancer Screening among Rural Populations: A Systematic Review. Int. J. Environ. Res. Public Health.

[14] Kale S., Hirani S., Vardhan S., Mishra A., Ghode D.B., Prasad R., Wanjari M. (2023). Addressing Cancer Disparities Through Community Engagement: Lessons and Best Practices. Cureus.

[15] Ras T., Adeleke O., Moodley J. (2024). The role of primary care practitioners in cancer control in South Africa: A systems-based case study. S. Afr. Health Rev..

[16] Tshabalala G., Blanchard C., Mmoledi K., Malope D., O’nEil D.S., Norris S.A., Joffe M., Dietrich J.J. (2023). A qualitative study to explore healthcare providers’ perspectives on barriers and enablers to early detection of breast and cervical cancers among women attending primary healthcare clinics in Johannesburg, South Africa. PLoS Glob. Public Health.

[17] Fereidouni Z., Dehghan Abnavi S., Ghanbari Z., Gashmard R., Zarepour F., Samani N.K., Sharma A.R., Ghasemi A. (2024). The Impact of Cancer on Mental Health and the Importance of Supportive Services. Galen Med. J..

[18] Puchalski C.M., Sbrana A., Ferrell B., Jafari N., King S., Balboni T., Miccinesi G., Vandenhoeck A., Silbermann M., Balducci L. (2019). Interprofessional spiritual care in oncology: A literature review. ESMO Open.

[19] Grossman C.H., Brooker J., Michael N., Kissane D. (2018). Death anxiety interventions in patients with advanced cancer: A systematic review. Palliat. Med..

[20] O’Donovan J., O’Donovan C., Nagraj S. (2019). The role of community health workers in cervical cancer screening in low-income and middle-income countries: A systematic scoping review of the literature. BMJ Glob. Health.

[21] Brockhoven F., Raphael M., Currier J., Jäderholm C., Mody P., Shannon J., Starling B., Turner-Uaandja H., Pashayan N., Arteaga I. (2023). REPRESENT recommendations: Improving inclusion and trust in cancer early detection research. Br. J. Cancer.

[22] Moshabela M., Bukenya D., Darong G., Wamoyi J., McLean E., Skovdal M., Ddaaki W., Ondeng’e K., Bonnington O., Seeley J. (2017). Traditional healers, faith healers and medical practitioners: The contribution of medical pluralism to bottlenecks along the cascade of care for HIV/AIDS in Eastern and Southern Africa. Sex. Transm. Infect..

[23] Galvin M., Chiwaye L., Moolla A. (2024). Religious and Medical Pluralism Among Traditional Healers in Johannesburg, South Africa. J. Relig. Health.

[24] Zuma T., Wight D., Rochat T., Moshabela M. (2018). Navigating Multiple Sources of Healing in the Context of HIV/AIDS and Wide Availability of Antiretroviral Treatment: A Qualitative Study of Community Participants’ Perceptions and Experiences in Rural South Africa. Front. Public Health.

[25] Msoka E.F., Dwarampudi S., Billings R., Stone R.J., Mwageni R.E., Beavers A., Mmbaga B.T., Gutnik L. (2025). The role of traditional healers along the cancer care continuum in Sub-Saharan Africa: A scoping review. Arch. Public Health.

[26] O’Donovan J., Newcomb A., MacRae M.C., Vieira D., Onyilofor C., Ginsburg O. (2020). Community health workers and early detection of breast cancer in low-income and middle-income countries: A systematic scoping review of the literature. BMJ Glob. Health.

[27] Williams L.B., Looney S.W., Joshua T., McCall A., Tingen M.S. (2021). Promoting Community Awareness of Lung Cancer Screening Among Disparate Populations: Results of the cancer-Community Awareness Access Research and Education Project. Cancer Nurs..

[28] Albashir G., Sojourner S., Vernon M., Moore J., Looney S., Tingen M. (2021). Abstract 2550: Participant’s satisfaction with the cancer community awareness access research and education (C-CARE) project at urban and rural sites. Cancer Res..

[29] Vernon M.M., Sojourner S., Looney S., Tingen M. (2020). Abstract PO-024: Comparison of urban and rural participants in the cancer-Community Awareness Access Research and Education (c-CARE) Project. Cancer Epidemiol. Biomark. Prev..

[30] Escoffery C., Lebow-Skelley E., Haardoerfer R., Boing E., Udelson H., Wood R., Hartman M., Fernandez M.E., Mullen P.D. (2018). A systematic review of adaptations of evidence-based public health interventions globally. Implement. Sci..

[31] Buthelezi U., Lubuzo B., Tingen M., Chiya H., Gigaba S., Goieman B., Ramotshela S., Luvuno Z. (2026). Adapting a U.S. Cancer Education Programme for South Africa: A Participatory, Culturally Tailored Approach Using Card’s Seven-Step Adaptation Framework. Afr. J. Prim. Health Care Fam. Med..

[32] Koshiaris C., Oke J., Abel L., Nicholson B.D., Ramasamy K., Bruel A.V.D. (2018). Quantifying Intervals to Diagnosis in Myeloma: A Systematic Review and Meta-Analysis, Haematology (Incl Blood Transfusion). BMJ Open.

[33] Pooe A.M., Dlova A.N., Ntuli S.T. (2023). Medical practitioners’ knowledge and awareness of multiple myeloma at public hospitals, Gauteng, South Africa. S. Afr. Fam. Pract..

[34] Card J.J., Solomon J., Cunningham S.D. (2011). How to adapt effective programs for use in new contexts. Health Promot. Pract..

[35] IBM Corp (2024). IBM SPSS Statistics for Windows.

[36] Fisher M.J., Marshall A.P., Mitchell M. (2011). Testing differences in proportions. Aust. Crit. Care.

[37] Koo M.M., Unger-Saldaña K., Mwaka A.D., Corbex M., Ginsburg O., Walter F.M., Calanzani N., Moodley J., Rubin G.P., Lyratzopoulos G. (2021). Conceptual Framework to Guide Early Diagnosis Programs for Symptomatic Cancer as Part of Global Cancer Control. JCO Glob. Oncol..

[38] Akin-Odanye E.O., Husman A.J. (2021). Impact of stigma and stigma-focused interventions on screening and treatment outcomes in cancer patients. Ecancermedicalscience.

[39] Beeken R.J., Simon A.E., von Wagner C., Whitaker K.L., Wardle J. (2011). Cancer fatalism: Deterring early presentation and increasing social inequalities?. Cancer Epidemiol. Biomark. Prev..

[40] Mwobobia J.M., Sardana S., Abouelella D., Posani S., Ledbetter L., Graton M., Osazuwa-Peters N., Knettel B.A. (2025). Experiences of Cancer-Related Stigma in Africa: A Scoping Review. Int. J. Cancer.

[41] Radbruch L., De Lima L., Knaul F., Wenk R., Ali Z., Bhatnaghar S., Blanchard C., Bruera E., Buitrago R., Burla C. (2020). Redefining Palliative Care—A New Consensus-Based Definition. J. Pain Symptom Manag..

[42] van Heerden E.M., Jenkins L.S. (2022). The role of community health workers in palliative care in a rural subdistrict in South Africa. Afr. J. Prim. Health Care Fam. Med..

[43] Campbell L., Amin N. (2014). A qualitative study: Potential benefits and challenges of traditional healers in providing aspects of palliative care in rural South Africa. Rural. Remote Health.

[44] Ratshikana M., Ballot D., Myezwa H., Galantino M.L., Pilusa S. (2025). Spiritual needs, practices and associated factors among patients with cancer at two teaching hospitals. Afr. J. Prim. Health Care Fam. Med..

