# Supplementary material for: Improving Cancer Awareness and Knowledge in Johannesburg and iLembe Districts Through a Tailored Community-Based Educational Intervention: A Pilot Study
Source: Int J Environ Res Public Health. 2026 Jul 3;23(7):871. doi: 10.3390/ijerph23070871 (PMC13410355; doi:10.3390/ijerph23070871)
Supplement: Supplementary file 1 [file ijerph-23-00871-s001.zip › ijerph-4172744-supplementary.pdf]

Subject ID

Adapted cancer-Community Awareness Access Research and Education II (c-CARE II) Baseline Survey

Georgia Cancer Center, AFIA TAI, Genius Quality, Move for Transformation Ministries and UKZN
Martha S. Tingen, PhD[1], RN, FAAN, Bridgette J. Goeieman. MBChB[2], and Hlolisile Chiya, PhD[3]

1. Tel: 001 706-721-8418; e-mail: mtingen@augusta.edu
2. Tel: O714577188; e-mail: Bridgette.goeieman@righttocare.org
3. Tel: 076 317 9187; ChiyaW@ukzn.ac.za

Table S1. Baseline Survey (Pre- and Post) Questionnaire

Directions: Complete the survey if you are at least age 21. Fill in the blanks or place a check (√) in the box that most closely describes you. This survey is anonymous – Please do not write your name on it.

Section I – A little about you

Today's date: ____/ ___/ ____ (dd/mm/yyyy)

1. What is your age? __________

2. Gender
1.Male
2. Female
3. Other

3.Your current role:

4. Your race:
1. Black African
2. Coloured
3. Indian/Asian
4. White
5. Other / Prefer not to specify

5. Your religious preference:
1. Christian
2. Hindu
3. Muslim
4. African Traditional Religion
5. Other
6.No preference

6. Your highest level of education:
1. Illiterate
2. Primary
3. Secondary
4. Tertiary

7. Employment status:
1. Student (full or part-time)
2. Self-employed
3. Work Full-Time (36 hrs or more/week)
4. Work Part-Time (less than 36 hrs/week)
5. Retired and not employed.
6. Unemployed
7. Grant

8. Have you ever been exposed to any type of cancer training?
1. Yes
0. No
If yes, for how many days was it conducted)

9. What is your first point of contact when you are sick?

1. Public health facility
2. Private health facility
3. Traditional Health Practitioner
4. Spiritual healer
5. Other specify

Section II – What do you know about cancer?

Directions: **Check the best answer.** **Select only one answer please.**

1. Choose the statement that is correct about **cancer risk factors**. Risk factors are grouped as:
1. Genetic and family history.
2. Behavioral, environmental, genetic and biological.
3. Biological, age, sex, and race.
4. Behavioral, smoking, and occupation.

2. Have you ever heard about **Lung Cancer**?
1. Yes
0. No– ***Skip to the next section***

How did you receive information about **Lung Cancer**?
1. Healthcare provider
2. Family or friend
3. Online sources (WebMD, etc.)
4. Not sure/Do not recall

3. Which one of the following is the recommended screening test for **Lung Cancer**?
1. X-ray
2. CT (CAT) scan
3. MRI
4. Blood work
5. Don't know

4. Which one of the following cigarette ingredients <u>does not</u> cause **Lung Cancer**?
1. Carbon monoxide
2. Tar
3. Nicotine
4. Methane
5. Don't know

5. Choose the one statement that is correct about **Lung Cancer** screening:
1. Doctors have not made a lung cancer screening test yet.
2. Lung cancer screening has been shown to improve survival.
3. Lung cancer screening has many risks that outweigh the benefits of screening.
4. Not enough information is known about the benefits of lung cancer screening.

6. Please indicate your response about the **harmfulness of electronic cigarettes/e-cigs**; Compared to smoking cigarettes, would you say that electronic cigarettes/e-cigs are:
1. Much less harmful
2. Less harmful
3. Just as harmful
4. Not harmful at all
5. Don't know

7. Choose the statement about the signs of **Lung Cancer** that is NOT correct.

1. Cough, bloody phlegm, chest pain and voice changes.
2. Bloody phlegm, weight loss, fatigue, and voice changes.
3. Trouble breathing, weight gain, cough, and voice changes.
4. Trouble swallowing, frequent pneumonia, cough, and fatigue.

8. Have you ever heard of **Cervical Cancer** or **Cervical Cancer** screening?

1. Yes
0. No– ***Skip to the next section***

9. Have you ever heard of human papillomavirus (HPV) or the HPV vaccine?

1.Yes
0. No

10. Who can be vaccinated under the department of health program?

1. All males and females
2. Girls only any age
3. Girls of the age of 10 years in grade five
4. Boys only

11. Cervical Cancer is preventable.

1. True
0. False

12. Which of the following are risk factors (causes) of **Cervical Cancer**? (Select all that apply.)

1. Family members with cervical cancer
2. Having human papilloma virus (HPV) or HIV
3. Smoking/tobacco use
4. Use of oral contraception
5. Having many different sexual partners.
6. Giving birth to many babies.

13. Which of the following are signs/symptoms of **Cervical Cancer?** (Select all that apply.)

1. Irregular vaginal bleeding
2. Ongoing lower back pain
3. Abnormal or smelly vaginal discharge
4. Pain during sex
5. Heavier or longer menstrual periods than normal
6. Vaginal bleeding after menopause
7. Bloody stools
8. Unexplained weight loss

14. Have you ever heard about **Breast Cancer?**

1. Yes
0. No– ***Skip to the next section.***

How did you receive information about **Breast Cancer?**

1. Healthcare provider
2. Family or friend
3. Online sources (Internet/Media, etc.)
4. Previous training
5. Not sure/Don't recall.

15.Which one of the following is the recommended screening test for **Breast Cancer**?

1. CT (CAT) scan
2. Mammogram
3. MRI
4. Blood work
5. Unsure/Do not know.

16. Choose the statement that is correct about **Breast Cancer** screening.

1. Breast cancer screening cannot detect early breast cancer.
2. Breast cancer screening has been shown to improve survival.
3. Breast cancer screening has many risks that outweigh the benefits of screening.
4. Not enough information is known about the benefits of breast cancer screening.

17. Choose the statement about the signs of **Breast Cancer** that is NOT correct.

1. New lump in the breast or underarm, any change in size or shape of the breast.
2. Thickening or swelling of part of the breast, nipple discharge other than breast milk.
3. Tingling and/or itching in any area of the breast.
4. Pain, redness, or flaky skin in the nipple area or any area of the breast.

18. Have you ever recommended someone to get screened for **Breast Cancer**?

1. Yes
0. No

19. Have you ever heard about **Prostate Cancer**?

1. Yes
0. No– ***Skip to the next section.***

How did you receive information about **Prostate Cancer**?

1. Healthcare provider
2. Family or friend
3. Online sources (Internet/media, etc.)
4. Previous training
5. Not sure/Don't recall.

20, Which one of the following is a common screening test for **Prostate Cancer**?

1. CT (CAT) scan

2. Digital Rectal Exam (DRE)
3. Stress test
4. Unsure/Do not know

21. Choose the statement that is correct about **Prostate Cancer** screening.
1. Prostate cancer screening cannot detect early prostate cancer.
2. Prostate cancer screening has been shown to improve survival.
3. Prostate cancer screening has too many risks that outweigh the benefits of screening.
4. Not enough information is known about the benefits of prostate cancer screening.

22. Choose the statement about the signs/symptoms of **Prostate Cancer** that is NOT correct.
1. Difficult or painful urination, or blood in the urine.
2. Slow or weak urinary stream, or the need to urinate more often, especially at night.
3. Frequent/excessive sweating in groin area.
4. Bone pain in hips, back, or chest.

23. My healthcare provider has recommended that I get screened for **Prostate Cancer**.
1. Yes
0. No

24. Have you ever heard about **Multiple Myeloma**?
1. Yes
0. No– ***Skip to the next section.***

How did you receive information about **Multiple Myeloma**?
1. Healthcare provider
2. Family
3. Friend
4. Online sources (Internet/media, etc.)
5. Previous training
6. Not sure/Don't recall.

25. Which tests can help identify **Multiple Myeloma**? (Select all that apply.)
1. CT (CAT) scan/ X-Rays
2. Blood work
3. Urine
4. Biopsies
5. Unsure/Do not know

26. The term C.R.A.B. stands for what, as it relates to **Multiple Myeloma** symptoms?
1. Cholesterol, Redness, Abscess, Bone Pain
2. Cold, Redness, Abnormal Platelets, Bruising
3. Calcium, Redness, Anemia, Bone Fracture
4. Calcium, Renal Failure, Anemia, Bone Lesions

27. Have you ever recommended someone to get screened for **Multiple Myeloma**?
1. Yes
0. No

Section III – How do you feel about cancer?

Directions: Read each statement. Check (√) the box to the right that best describes you.

1. = Agree 2. =Disagree 3. = Not sure 4.= Don't Know

Statement	1.	2.	3.	4.
1. Most people are afraid to do a cancer screening because they think they will find out that they have cancer.				
2. If I get cancer, it was meant to be.				
3. There is not much that a person can do to lower their chances of getting cancer.				
4. Getting checked regularly for cancer helps find cancer when it's easy to treat.				
5. Most people are comfortable discussing their medical history.				
6. When people think of cancer, they automatically think of death.				
7. Most people prefer not to know if they have cancer.				
8. Exposing cancer to air during surgery causes it to spread faster.				
9. It seems like everything causes cancer.				

Section IV – What do you know about Palliative Care, Spiritual Care, and Mental Health?

Directions: Select the best answer or answers.

1. Which of the following describes **Palliative Care**: (Select all that apply)
1. Care for people who are dying only
2. Care for people who have serious illnesses
3. Care for patients and their families
4. Care that covers the physical, social, emotional, and spiritual issues that patients face
5. Care for the child only

2. The team that provides **Palliative Care** includes: (Select all that apply)
1. Doctors
2. Nurses
3. Social Workers and Social Auxiliary Workers
4. Chaplains and Spiritual Counsellors
5. Community Health Care workers
6. Don't know

3. Locations where **Palliative Care** can be provided are:
1. In the hospital only
2. In the hospital, clinic and the home of the patient
3. Don't know

4. The role of a **community health care worker in Palliative Care** includes: (Select all that apply)
1. Treating pain and other distressing symptoms
2. Identifying patients who need palliative care
3. Referring patients to a health care worker
4. Finding out if patients are taking medication as prescribed
5. Don't know

5. Which of the following statements do you associate with **Spirituality**? (Select all that apply)
1. Is important to cancer patients
2. Improves the quality of life of cancer patients
3. The root cause of most difficulties people face can be spiritual
4. If not addressed can result in suffering
5. Cancer patients have spiritual needs

6. Which of the following statements about **Spiritual Care** are true? (Select all that apply)
1. Every patient has a right to receive spiritual care.
2. It helps cancer patients who are in pain.
3. Community health care workers can pray with any patient when asked.
4. It is part of holistic patient centred care.
5. May help the patient find meaning in life.

7. **Spiritual Care** practices include the following: (Select all that apply)
1. Listening
2. Providing space for meditation and prayer.
3. Screening for spiritual needs.
4. Referring to a spiritual counsellor/chaplain.
5. Identifying patient's resources for hope.

8. Which of the following statements is <u>incorrect</u> about **Mental Health** screening:

1. Finds and prevents diseases.
2. Can only be done by doctors in hospitals.
3. Provides information about the community members lifestyles.
4. Identifies mental health problems early.

9. Which one of these is NOT a sign of **mental illness**?

1. They are unable to cope with the normal stresses of life.
2. They are able to work productively.
3. Their behavior negatively affects those around them.
4. Their eating habits may change.

10. Tracing and retention in care is not applicable to **Mental Health** patients.

1. True
0. False

11. Which of the following statements about referral for **Mental Health** care is/are <u>correct</u>?

1. The client must have been screened by the CHW or other health worker.
2. The client must show a clear understanding of why there is a need for referral.
3. The client must have indicated that they have mental health issues that have a negative impact on daily activities.
4. All of the above

Thank you for completing this survey.
